# Experiences and lessons learned for delivery of micronutrient powders interventions

**DOI:** 10.1111/mcn.12495

**Published:** 2017-09-29

**Authors:** Ietje Reerink, Sorrel ML Namaste, Alia Poonawala, Christina Nyhus Dhillon, Nancy Aburto, Deepika Chaudhery, Hou Kroeun, Marcia Griffiths, Mohammad Raisul Haque, Anabelle Bonvecchio, Maria Elena Jefferds, Rahul Rawat

**Affiliations:** ^1^ Independent Consultant Antananarivo Madagascar; ^2^ Strengthening Partnerships, Results, and Innovations in Nutrition Globally Arlington Virginia USA; ^3^ Helen Keller International Washington District of Columbia USA; ^4^ Global Alliance for Improved Nutrition Geneva Switzerland; ^5^ Independent Consultant Geneva Switzerland; ^6^ World Food Programme Rome Italy; ^7^ Micronutrient Initiative New Delhi India; ^8^ Helen Keller International Phnom Penh Cambodia; ^9^ The Manoff Group Washington District of Columbia USA; ^10^ BRAC Dhaka Bangladesh; ^11^ Instituto Nacional de Salud Publica Cuernavaca Mexico; ^12^ Nutrition Branch Centers for Disease Control and Prevention Atlanta Georgia USA; ^13^ International Food Policy Research Institute Dakar Senegal; ^14^ Bill and Melinda Gates Foundation Seattle Washington USA

**Keywords:** behaviour, communication, complementary feeding, iron deficiency anaemia, micronutrients, programming

## Abstract

An effective delivery strategy coupled with relevant social and behaviour change communication (SBCC) have been identified as central to the implementation of micronutrient powders (MNP) interventions, but there has been limited documentation of what works. Under the auspices of “The Micronutrient Powders Consultation: Lessons Learned for Operational Guidance,” three working groups were formed to summarize experiences and lessons across countries regarding MNP interventions for young children. This paper focuses on programmatic experiences related to MNP delivery (models, platforms, and channels), SBCC, and training. Methods included a review of published and grey literature, interviews with key informants, and deliberations throughout the consultation process. We found that most countries distributed MNP free of charge via the health sector, although distribution through other platforms and using subsidized fee for product or mixed payment models have also been used. Community‐based distribution channels have generally shown higher coverage and when part of an infant and young child feeding approach, may provide additional benefit given their complementarity. SBCC for MNP has worked best when focused on meeting the MNP behavioural objectives (appropriate use, intake adherence, and related infant and young child feeding behaviours). Programmers have learned that reincorporating SBCC and training throughout the intervention life cycle has allowed for much needed adaptations. Diverse experiences delivering MNP exist, and although no one‐size‐fits‐all approach emerged, well‐established delivery platforms, community involvement, and SBCC‐centred designs tended to have more success. Much still needs to be learned on MNP delivery, and we propose a set of implementation research questions that require further investigation.

## 1 INTRODUCTION

Despite concerted efforts, there has been limited success in reducing the high prevalence of anaemia in low‐ and middle‐income countries, with only a 4% decrease in the prevalence among children over more than a decade (Stevens et al., 2013). Micronutrient powders (MNP), a mixture of vitamins and minerals, enclosed in single‐dose sachets that are stirred into a child's portion of food immediately before consumption, are one of the few available interventions to address the nutritional causes of anaemia and fill important nutrient gaps in children (De‐Regil, Suchdev, Vist, Walleser, & Peña‐Rosas, 2013). Although the efficacy of MNP has clearly been demonstrated for improving iron status and reducing anaemia and their use is recommended by the World Health Organization (World Health Organization, 2011, 2016), effective delivery and long‐term sustainability of MNP interventions remain a complex challenge.

MNP interventions have been implemented in a wide range of countries. In just 4 years (2011–2014), the number of countries implementing MNP interventions to any target group doubled from 22 to 50, with 59 interventions globally (more than one intervention exists in some countries, and many are pilots) according to the most recent United Nations Children's Fund (UNICEF) NutriDash Global Report (2015). Despite this, only a small fraction of the 34 million children in the highest burden countries are targeted for this intervention, and globally, less than 5% of all children with anaemia receive MNP (Bahl, Toro, Qureshi, & Shaw, 2013). An MNP delivery strategy that optimizes MNP accessibility, coupled with social behavioural change communication (SBCC) and adequate training to support appropriate MNP use and intake adherence, is instrumental to successful MNP interventions. In fact, limited implementation of behaviour change activities was identified as a leading reason for the current gap between planned targets and children reached (UNICEF, 2015). As countries continue to design and scale up MNP interventions, there is an opportunity to reflect on what we have learned so far.

This paper is part of a series commissioned by the United States Agency for International Development (USAID) through the Strengthening Partnerships, Results, and Innovations in Nutrition Globally (SPRING) project to document experiences in planning, implementing, and monitoring MNP interventions focused on young children and interpret implications for programs globally. This paper examines MNP implementation, specifically delivery, SBCC, and training.

**Key messages**

- Different combinations of models, platforms, and channels have shown potential for micronutrient powders distribution, so program designers should feel encouraged to explore multiple ways to reach all population groups who could benefit.
- Delivery of micronutrient powders to young children is most common through infant and young child feeding programs because it focuses on the same target age and provides an opportunity for mutually reinforcing messaging and counselling. However, poorly implemented infant and young child feeding programs have posed a challenge to implementation across many contexts.
- Social and behaviour change communication activities have been successful in increasing micronutrient powders awareness, but how much this translates into appropriate use and intake adherence is inconsistent. The limited studies that have looked at the impact on associated infant and young child feeding behaviours have found improvements overall.
- Tailoring training for distributors has been necessary, with different needs depending on the delivery model (free, subsidized, or full cost) and delivery channel(s).

## 2 METHODS

A consultative group consisting of 49 practitioners with knowledge in the implementation of MNP interventions was formed. The process is described in the executive summary of this series (Nyhus Dhillon et al., 2017). Briefly, under the auspices of “The Micronutrient Powders Consultation Lessons Learned for Operational Guidance,” three working groups (WGs) were established: planning and supply (WG1); delivery, social and behaviour change communication, and training (WG2); and monitoring, process evaluation, and supportive supervision for continual programme improvement (WG3). The focus of the consultation was to review interventions that were fairly well established and scaled, targeting children 6–23 months of age. However, as the consultative process unfolded, learnings from pilots and programmes with a wider target age (up to 59 months of age) were included, as well as some relevant lessons from emergency settings.

Each WG was charged with synthesizing available evidence from programmatic settings. The outcomes of this effort are presented in this paper for WG2 and elsewhere in this series for WG1 (Schauer, et al., 2017) and WG3 (Vossenaar et al., 2017). WG2 consisted of a chair (RR) and 13 participants working for governmental institutions, multilateral and international organizations, universities, as well as independent consultants. WG members were based in Bangladesh, Cambodia, India, Madagascar, Mexico, Senegal, and the United States. WG2 participated in a year‐long (July 2015–July 2016) consultative process. It held three teleconferences to define the scope of the WG topic, participated in a meeting on October 19 and 20, 2015, in Washington D.C., United States, exchanged emails, conducted key informant interviews, and reviewed literature.

The WG obtained primary data from key informants identified using purposive and snowball sampling (Table 1). Key informants either completed a questionnaire or were interviewed using the same structured questionnaire (Supporting Information S1). Follow‐up with key informants to confirm data and seek additional information was performed as necessary. WG members involved in implementation also completed questionnaires or were interviewed. Data were analysed by collating the information into a spreadsheet and identifying relevant information. We also identified key informants to provide information for case studies to take a more in‐depth look at context‐specific learning. Key informants provided expert opinion as part of their professional capacity and regular public health practice. Thus, the activities involved in the consultative process did not meet the human subjects research definition and were considered exempt by the John Snow, Inc. Institutional Review Board. Interview participants were told that their names would be confidential in all reports and manuscripts and that any information gathered would be summarized in manuscripts submitted for peer review publication.

**Table 1:** Characteristics of key informants^a^

Key informant number	Country(ies) of work^b^	Role of informant	Scale of programme^c^	Data collection method	Date of interview
1	Kyrgyzstan	TA Provider	National	Interview Questionnaire	September 24, 2015 October 10, 2015
2	Indonesia	Implementer	Pilot	Questionnaire	October 2, 2015
3	Tanzania	Implementer	Subnational	Questionnaire	October 12, 2015
4	Bangladesh, Bolivia, Uganda	TA Provider	National, pilot	Questionnaire	October 13, 2015
5	Cambodia	Implementer	Pilot	Questionnaire	October 14, 2015
6	Bangladesh	Implementer	National	Interview	October 19, 2015
7	Lao PDR	TA provider	Pilot	Interview	December 15, 2015
8	Bangladesh, Mexico	TA provider	National	Interview	December 18, 2015
9	Madagascar	Implementer	Pilot	Interview Case study	December 21, 2015 May 29, 2016
10	Kenya, Vietnam	TA provider	Pilot	Interview	February 4, 2016
11	Mexico	TA Provider	National	Case study	July 12, 2016
12	Nepal	TA Provider	Pilot	Case study	July 14, 2016

a TA, technical assistance.

b Defined by the primary countries for which key informant provided experiences and learning.

c Defined by the stage of the intervention for which key informant provided experiences and learning.

The WG obtained secondary data from a systematic search of peer‐reviewed and grey literature. The search inclusion criteria were implementation learning on MNP from database inception through December 2015 and included a screening of abstracts, along with full texts when required, as described in more detail in the executive summary of this series (Nyhus Dhillon et al., 2017). A broad interpretation of relevance was applied when selecting literature to maximize the potential secondary data.

This paper is divided into three sections: delivery, SBCC, and training. Within the delivery strategy section, three distinct programme components are considered: delivery model(s), platform(s), and channel(s). We refer to the *model* as the cost of MNP to the consumer along a spectrum from free to subsidized to full cost, as well as mixed where price differentials for the product coexist as part of the same programme. The *platform* is defined as the existing programme, system, or structure used to deliver MNP and has been divided by health sector and nonhealth sector. The *channel* is the frontline distributor or mode used to deliver MNP. We chose not to use the terms *private* or *public* when defining the delivery strategy because interventions that combine elements of each are increasingly being designed. The delivery section in this paper is divided into common groupings of models and platforms followed by types of delivery channels and MNP schedules. Sections on SBCC and training follow.

Throughout this paper, we use programme metrics when they are available to compare implementation experiences. Success metrics from pilots and longer term MNP interventions were included, but efficacy trials were excluded. We used broad programme metrics definitions to allow us to capture data across interventions. We defined these metrics as *coverage* (“currently receiving or consuming MNP,” alternatively, “ever received/purchased or consumed” was used when this was the only available data and noted as such), *appropriate use* (preparing and consuming MNP as directed), and *intake adherence* (generally was “the percent of children reporting consumption of a given amount of sachets” and the denominator was either “all children” or “children receiving or consuming MNP” and noted as such). The denominator for each metric was based on the intervention target population (e.g., geographic scope and age) as defined by the programme.

Because there is no agreed upon threshold for effective programme performance, we selected a threshold of >70% coverage and/or intake adherence on the basis of experiences from vitamin A supplementation and IYCF programmes. The coverage target for vitamin A supplementation is 90% nationally and 80% (at a minimum) in districts, but countries are achieving an average coverage of 70% (UNICEF, 2007; WHO, 2012). There is currently no agreed upon target for the coverage of IYCF nutrition counselling for mothers of children 6–23, and data on the actual coverage were not available in most countries (UNICEF, 2015). Therefore, we considered IYCF practices as a proxy indicator, which showed 66% of children were introduced to solid, semisolid, or soft foods at 6–8 months of age, 11% of children 6–23 months of age received a minimum acceptable diet, and 49% of children were breastfed at 2 years of age in the least developed countries (UNICEF, 2016). When selecting the MNP coverage target, we took into consideration that vitamin A supplementation requires less behavioural change than an MNP intervention and, thus, it was not realistic to select a target of 90%. However, we did not select a target as low as the current coverage of IYCF practices to avoid the selection of a target that may not result in nutritional impact. It should also be noted that intake adherence is downstream from coverage, and thus, the intake adherence figures may reflect both caregivers' adherence and other factors such as resupply, as discussed in the planning paper of this series (Schauer et al., 2017).

The findings from this review are presented as a series of statements that relate to current practice, followed by details of the findings from countries on which these statements are founded. This analysis is not designed to provide results from any individual country. Terms and working definitions for the content of this paper, defined based on literature and key informants, are presented in Box 1. The authors acknowledge that other definitions may apply outside the context of this paper.

**Box 1. Definitions of terms used in programmatic research by working group 2 (WG2): Delivery, Social and Behaviour Change Communication, and Training^a^**

**Appropriate use of MNP**: preparing and consuming MNP as directed (e.g., mixing of no more than one sachet per day into semisolid lukewarm foods [at optimal viscosity] designated only for the targeted individual and consumed within 30 min of feeding).
**Communication source:** means used to communicate with specific audiences. Four broad categories of channels are interpersonal (one on one or in small groups); traditional media (e.g., street theatre); mass media (TV, radio, print, and large events); and social media (websites, social networks, messaging platforms, and blogs).
**Coverage of MNP:** percent of target population who have received/consumed/purchased MNP in a specific time period.
**Delivery channel:** distributor or mode through which an intervention is delivered (e.g., health facility workers or community health workers, pharmacists, commercial retailers, and agriculture extension workers).
**Delivery model:** type of public and/or private sector approach used as defined by the cost to the consumer (e.g., free, subsidized, or full cost).
**Delivery platform:** existing programme, system, or structure that can be used to deliver an intervention or a package of interventions (e.g., agriculture, health, social protection, early child development and education, and markets).
**Fixed intake schedule of MNP:** prescribed MNP intake schedule for the caregiver to follow (e.g., daily, every other day, and once a week).
**Flexible intake schedule of MNP:** recommended number of MNP given at will by caregiver to the child during a given time period but not more than one a day.
**Infant and young child feeding programme:** support practices of feeding infants and young children; examples of key recommended practices include (a) early initiation of breastfeeding within 1 hour of birth; (b) exclusive breastfeeding for the first 6 months of life; and (c) introduction of nutritionally adequate and safe complementary (solid) foods at 6 months together with continued breastfeeding up to 2 years of age or beyond (WHO & UNICEF, 2003).
**Intake adherence of MNP:** consumption of a minimum amount of MNP sachets over a recommended time period.
**Mixed model:** Hybrid of full cost, subsidized, or free model as part of the same intervention.
**SBCC interventions:** a research based, consultative process that uses communication to promote and facilitate behaviour change and support the requisite social change for the purpose of improving health outcomes (The Manoff Group, 2012).

^a^MNP, micronutrient powders; SBCC, social and behavior change.

## 3 RESULTS

Sixty‐six peer‐reviewed articles, 16 guidance documents, and 45 programme reports or conference presentations with information on programme implementation experiences were identified and reviewed (Nyhus Dhillon et al., 2017). Thirty documents were identified as relevant for delivery, SBCC, and training. Twelve key informants were interviewed, completed a questionnaire, or provided a case study (Table 1). Lessons from 14 countries in all six WHO geographic regions were included, some with multiple experiences with MNP pilots and programmes; case studies from Nepal, Mexico, and Madagascar were included to illustrate key experiences on the topics of this paper.

### 3.1 Delivery strategies

Using the definitions for delivery strategy defined in Section 2, we found that countries have adopted a wide variety of MNP delivery strategies. Pros and cons of different delivery strategies were synthesized across key informant interviews and are distilled in Table 2. Coverage and intake adherence data obtained from grey and published literature are organized by delivery strategy in Table 3. Data identified were mainly from pilots and/or short‐term trials and thus may not be applicable at scale.

**Table 2:** Pros and cons of different MNP delivery models and platforms identified by key informants^a^

Model/Platform^b^	Pros	Cons
Free/Health Sector	• Cost sharing if high level of integration • Can be built into routine IYCF training or serve as impetuous to conduct IYCF trainings • Linkages to IYCF information, services and referrals • Potential for large‐scale implementation if built into national system	• May rely on substantial external funding • IYCF programmes are often weak and quality can be even more compromised at scale • Supply vulnerable to interruptions when health system has weak supply management • Reach may not be uniform (e.g., geographic areas, ethnic groups) • May overburden the national health system
Free/Nonhealth sector	• Often government funded and opportunities for cost sharing • May target vulnerable households that are more in need of intervention • Targeting vulnerable subpopulation makes scale‐up more feasible • High potential for delivery at scale	• Supply chain can be difficult to establish • Linkages to IYCF information, services and referrals may be lacking if nutrition is not a primary objective • IYCF training needed for nonhealth staff • Eligibility may exclude less vulnerable populations that still would benefit from intervention and may not target households with children <2 years • May overburden the national nonhealth sector system
Full cost/any platform	• Commercially funded • Lower likelihood of stock‐outs • Potential for easy access (e.g., retail channels) • Opportunity to use already at scale market platform	• Requires well‐developed commercial sector and substantial start‐up investments • Linkages to IYCF information, services and referrals often lacking • Training limited and/or poorer quality • Does not reach households that cannot afford to pay
Subsidized/any platform	• Potential to recover some programmatic costs to expand scale • Reaches populations between poorest and richest	• Often still relies on some level of external funding • Often requires specific sales training for MNP distributors
Mixed/any platform	• Potential to recover some costs to expand scale • Reaches population in segments of those willing and able to pay from those that need the product for free	• Often still relies on some level of external funding • Free MNP can undercut commercial sales i.e. MNP may leak from the free distribution points and be resold de‐motivating retailers • Leakage between MNP products at different prices (but may be overcome by brand differentiation) • Often requires specific sales training

a IYCF, infant and young child feeding; MNP, micronutrient powders.

b Model = cost to consumer ranging from free to full cost; Platform = programme, system, or structure used to deliver MNP; linkages to IYCF information, services, and referrals depend on delivery channel, and potential for scale depends on integration into a country's broader system

**Table 3:** Coverage and intake adherence for MNP interventions^a^

Country	Model	Platform	Channel	Stage	Access duration	Coverage	Coverage Definition	Intake adherence	Intake adherence definition	Design
Cambodia (Helen Keller International, 2015)	Free	Health	Facility + community^b^	Pilot	2 yr	76%	% of children 6–23 mo received MNP in the last mo of the programme	56%	% of surveyed caregivers who report child consumed 15 sachets per mo	Monitoring data (coverage) and endline survey (intake adherence)
Guatemala (Olney, Rawat, & Ruel, 2012)	Free	Health	Community	Programme	Ongoing	73%	% of children 6–23 mo consuming MNP in the last wk	NA		Cross sectional survey as part of external process evaluation
Kenya (GAIN, n.d.)	Free	Health	Facility	Pilot	12 mo	33%	% of children 6–23 mo receiving MNP in targeted area	NA		Kajiado county pilot design unclear
Kenya^c^ (GAIN, n.d.)	Free	Health	Facility + community	Pilot	6 mo	72%	% of children 6–59 mo reached out of the target population	NA		Nairobi city county pilot design unclear
Kyrgyzstan (CDC & UNICEF, 2013)	Free	Health	Facility + community	Programme	2 yr	74%	% of caretakers interviewed who say child is “currently” consuming MNP	NA		Follow‐up cross‐sectional survey
Mongolia (World Vision Mongolia, 2005)	Free	Health	Community	Pilot	2 yr	48%	% of children 6–35 mo consuming MNP at time of final survey	88%	% of children 6–35 mo taking MNP at final survey who had done so for 4 or more mo.	Endline cross‐sectional survey.
Nepal (Jefferds et al., 2015)	Free	Health	Community	Pilot	15 mo	83%	% of children ever obtaining a batch of 60 MNP	52%	% mother reported obtaining MNP ≥2 mo before date of interview	Two cross‐sectional survey in two pilot districts each
Nepal (Jefferds et al., 2015)	Free	Health	Facility	Pilot	15 mo	52%	% of children ever obtaining a batch of 60 MNP	35%	% mother reported obtaining MNP ≥2 mo before date of interview	Two cross‐sectional survey in two pilot districts each
Nigeria (Korenromp et al. 2015)	Free	Health	Maternal and child health days	Pilot within programme	6 mo	32%	% of children 6–59 mo who received MNP	51–69%	% of caretakers who received MNP reporting consumption of 2 or 3 sachets per wk at 1 mo post distribution	Cross‐sectional survey at distribution (coverage) and repeat survey of caregivers who received MNP 1 mo post distribution (intake adherence)
India^d^ (Hirve et al., 2013)	Free	ECD	Community	Programme	4 mo	NA		84%	% of children 6–59 mo who consumed 60 sachets over a 4 mo period	Compliance cards from monitoring data
Nicaragua (Lopez Boo et al., 2014)	Free	ECD	Community	Programme	2 mo	75%	% of children 6–59 mo in programme receiving MNP	70%	% of children receiving MNP who consumed “as recommended” (recommendation not reported)	Longitudinal data from a cost effectiveness study
Mexico (Bonvecchio & PROSPERA Program, 2016)	Free	Social Protection	Facility + community	Programme	Ongoing	93%	% of beneficiary children 6–59 mo old receiving 60 sachets of MNP in the last 2 mo	78% (urban) 81% (rural)	% of targeted beneficiary children consuming MNP daily, among those receiving	Monitoring data (coverage) cross‐sectional survey (intake adherence)
China^e^ (Sun et al., 2011)	Subsidized	Health	Facility	Pilot	8 mo	13%	% of caretakers who ever purchased MNP	95%	% of children who consumed the product at least 3× per wk, among those who purchased the product	Based on interviews of 226 caregivers
Madagascar^f^ (Ramalanjaona et al., 2015)	Subsidized^c^	Health	Facility + community	Pilot	18 mo	46%	% of caregivers of children 6–23 mo had purchased 1 box of MNP	1.8 mo	Average duration of use	Endline cross sectional survey
Vietnam (Nguyen et al., 2016)	Subsidized	Health	Facility	Pilot	6 mo	23%	% of caretakers who had ever given product to child	12%	% of caregivers reporting consumption of ≥3 sachets per child per wk (among total target population not just those giving product)	Cross‐sectional survey 5 mo after distribution started
Bangladesh^g^ (Rawat et al. 2013	Subsidized^c^	Commercial	Door‐to‐door	Programme	18 mo	17%	% of households reportedly purchasing MNP	NA		Mid‐term survey in half of evaluation districts
Bangladesh^g^ (Angdembe et al., 2015)	Subsidized^c^	Commercial	Door‐to‐door	Programme	Ongoing	NA		70%	% of total sachets consumed out of total number of days taken to consume sachets for all children 6–59 mo who used MNP in previous 60 days	Cross sectional survey
Ghana^h^ (Aaron et al., 2016)	Subsidized	Commercial	Retail shop (urban)	Pilot	1 yr	9%	% of children 6–23 mo given product at least once in last 7 days	NA		Cross‐sectional survey
Ghana^h^ (Aaron et al., 2016)	Subsidized	Commercial	Facility + community (rural)	Pilot	1 yr	86%	% of children 6–23 mo given product at least once in last 7 days	NA		Cross‐sectional survey
Kenya^i^ (Suchdev et al. 2013)	Subsidized	Commercial	Door‐to‐door	Pilot	4 yr	65% (2008) 35% (2009) 22% (2010)	% of household used MNP in the past seven days	3.2 sachets (2008) 1.6 sachets (2009) 1.1 sachets (2010)	Average intake of MNP sachets per wk among purchasing households	Cross‐sectional survey

a Countries are first organized by free and then subsidized model and within these categories by platform; ECD, early child development; MNP, micronutrient powder; mo, months; NA, not available; wk, weeks; yr, years.

b Includes either a facility + community or facility only arm.

c Mixed model (free and subsidized) but data only available for free delivery arm.

d Includes MNP provided at home and at ECD centres.

e MNP also included soybean powdered flour.

f Mixed model (subsidized but with price differential) but data only available for the lower subsidized price point.

g Mixed model (free and subsidized) but data only available for subsidized delivery arm.

h MNP also included macronutrients, lysine, and flavourings.

i The 2010 data were 18 months after study related marketing and household monitoring ended.

**Figure 1:**
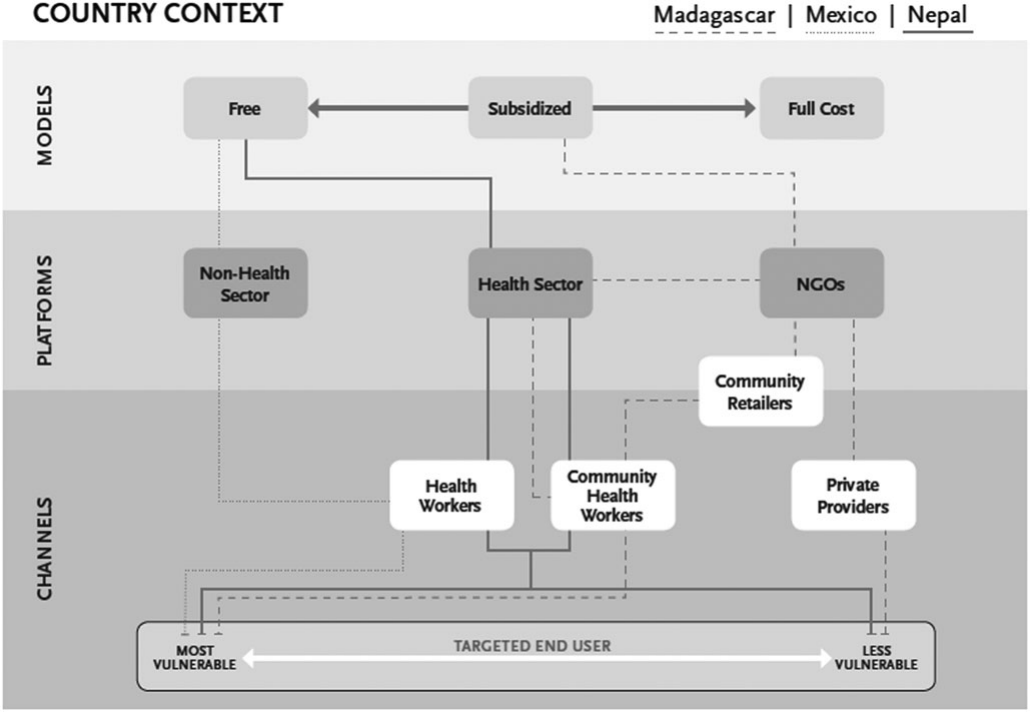
Case study examples of delivery strategies. Delivery strategies for three country case studies are presented. The delivery strategy for Madagascar is denoted with a dashed line, for Mexico with a dotted line, and for Nepal with a straight line. Models are presented as cost to the consumer ranging from *free* (as with Mexico and Nepal) to *subsidized* (as with Madagascar) to *full cost*. Platforms are presented as the programme, system, or structure used to deliver micronutrient powder (MNP) ranging from *health sector*, which generally include maternal and child services and/or programs for infant and young child feeding (as with Nepal), growth monitoring, maternal child health week distribution; *nonhealth sector*, which generally include small‐scale agriculture‐nutrition programs, early childhood development, and social protection programs (as with Mexico); and *nongovernmental organizations*, which are outside of the government system (as with Madagascar). Delivery channels are presented as the distributor or mode through which an intervention is delivered ranging from *health workers*, which generally include public sector physicians (as with Mexico), public sector nurses (as with Mexico), and other public health facility staff (as with Nepal); *community health workers*, which generally include paid community health workers or volunteer community health workers (as with Madagascar and Nepal); and *private sector providers*, which generally include private sector physicians (as with Madagascar), private sector nurses, and other private health facility staff. *Community retailers* can serve as the delivery channel by providing the micronutrient powders directly to consumer or serve as a supplier linking the platform to the community delivery channel provider (as with Madagascar). Based on information from key informants 9, 11, and 12.

Overall, we identified three of the most common types of delivery strategies on the basis of the literature review and key informant interviews. In order to illustrate these strategies, we used three case studies, one for each strategy, as depicted in Figure 1. Nepal represents the most common model (free model) delivered through the health platform via health facility workers and community health workers (CHWs) channels (Box 2) (Jefferds et al., 2015; KI 12). Mexico provides an example of a free model and nonhealth sector platform (i.e. MNP are provided to the beneficiaries of the country's social protection programme), although the MNP are delivered through the health sector via health worker channels (Box 3) (Bonvecchio & PROSPERA Program, 2016; KI 11). And, finally, Madagascar is an example of a mixed model delivered through the health and nongovernmental organization platform via a variety of distribution channels (Box 4)  (PSI Research Division, 2014; KI 9).

**Box 2: Nepal case study: Free public health sector delivery^a^^,^ b**

**Table:** 

Where	In six districts of Nepal (mountains, hills, and plains)
When	2010–2011 pilot
MNP delivery strategy	Model: Free MNP Platform: Health sector, integration with IYCF programme. Channel: Trained FCHV and/or HF staff promoting and providing the MNP to mothers. The health volunteers were the main media for SBCC on MNP use.
Target population	All children 6–23 months in six districts in an 18‐month pilot programme.
MNP schedule	Mothers were given 60 sachets and instructed to give their child one sachet daily until all sachets had been consumed and to return for an additional 60 sachets 6 months after the first batch had been received.
SBCC	Formative research was conducted to develop the SBCC component of the pilot, which included counselling, support groups, radio spots, brochures, and reminder cards. HF staff and FCHV received training on their role in delivery of the SBCC plan, which focused on building support for MNP, providing information and skills to increase their ability, opportunity and motivation towards improved IYCF practices, MNP use, active feeding, and hand washing and sanitation. There was an association having attended a mothers' group meeting or having hear about MNP on the radio and obtaining MNP (Jefferds et al., 2015).
Training	A training curriculum and job aids were developed to guide all trainings. A 4‐day master training of trainers helped develop facilitators to conduct training in districts with district level staff including community volunteers. At the start of the programme in each district, first the district leaders and HF in‐charges participated in 2 days of training on the programme. Then all HF staff participated in 2 days of training. Last, FCHV and community leaders participated in 4 days of training on the programme. Periodic refresher trainings and review meetings were conducted.
Lessons learned	A key programmatic challenge was the need to balance frequent contact with health workers—which was associated with higher intake in this pilot—with the risk of overstretching HF/FCHV staff. Supporting mothers to establish a routine for the child's MNP intake is a key strategy to support intake adherence regardless of the recommended intake schedule. The MNP intake schedule may have led to different levels of MNP intake adherence given that there was a 4‐month gap for mothers to re‐establish the use of MNP‐giving routine. Most of the barriers mentioned by mothers as reasons for not adhering to MNP could be addressed through regular information provision to resolve doubts and answer questions, consistent messaging about side effects and benefits, and using “fresh” reminders and prompts to counter dropout.

a FCHV, female community health volunteer; HF, health facility; IYCF, infant and young child feeding; MNP, micronutrient powders; SBCC, social behavior change communication.

b Based on information from key informant 12.

**Box 3: Mexico case study: Free nonhealth sector delivery^a^^,^ b**

**Table:** 

Where	At national scale in 32 states. The training and communication strategy is being implemented in 29 states. Three states were temporarily excluded because they were selected as control states for the training impact evaluation.
When	2014—ongoing programme
MNP delivery strategy	Model: Free to beneficiaries of the *Prospera* (SP) programme Platform: Public health system's primary care services Channel: Physicians and nurses from health centres
Target population	Approximately 6.1 million families are part of *Prospera* (targets are created every 2 months and based on monitoring data) 1,230,360 children 6–59 months (out of 1,328,646) received the supplements during the period January to February 2016.
MNP schedule	Daily doses year‐round. Mothers receive a bimonthly supply of the product (one box of 60 sachets) during their children's routine medical check‐ups. In rural areas, children 6–12 months receive a fortified porridge and children 12–24 months fortified milk in addition to MNP.
SBCC	A social marketing strategy was developed using formative research with health workers, mothers, fathers, and community leaders. An IYCF strategy was designed including MNP promotion. The strategy was piloted and feedback provided to improve it before scaling up nationally. The strategy is built on existing human resources; staff/infrastructure; and routine activities from within the cash transfer programme. SBCC messaging was standardized for consistency at all levels. Trained physicians/ nurses provide counselling and health promoters conduct workshops about IYCF and MNP use. Programme designed for community volunteers to make home visits to encourage the use of the MNP, help mothers cope with children rejecting food prepared with MNP, and manage side effects, but it is not fully implemented yet. Due to budgetary constraints, mass communication (radio, banners, videos, and TV spots) was funded only in one state targeting the indigenous population.
Training	After an unsuccessful initial traditional cascade training, the government and its partners developed a mixed training model on healthy pregnancy, IYCF, and MNP, which included in‐class and computer‐based courses to increase the reach to more health personnel and their motivation to complete the training. The training of trainers was a 1‐day, face‐to‐face course plus 45 hr for physician and nurses and 35 hr for health promoters that can be taken in a period of 5 weeks online. For health workers (frontline physicians, nurses, nutritionists, and health promoters) training was 1‐day, face‐to‐face, plus a 3‐week offline course. The training included IYCF, use of MNP, and ways to promote MNP. By June 2016, more than 48,000 health workers have been trained, more than half of the 75,000 goal.
Lessons learned	The programme was designed for scale at the onset and included nutrition specific objectives. Developing an enabling policy environment was key to ensure commitment for scaling up and to securing funding. Political pressure for rapid scale up precluded the timely implementation of the SBCC prior to MNP distribution. Integration within the conditional cash transfer programme and the health system was key to reaching a large population and ensuring implementation funding, although funding was not enough for a longer, in‐person training and the use of mass media. Funding constraints may be overcome by incorporating private funding mechanisms that do not represent conflict of interest to strengthen community mobilization efforts and incorporate mass media.

a IYCF, infant and young child feeding; MNP, micronutrient powders; SBCC, social behavior change communication

b Based on information from key informant 11.

**Box 4: Madagascar case study: Mixed health sector delivery^a^^,^ b**

**Table:** 

Where	Two rural and two urban areas of Madagascar
When	2013–2014 pilot, the programme is now being scaled up
MNP delivery strategy	Model: Mixed, social marketing for the rural areas and social franchising for urban areas Platform: Health sector; integration with IYCF programme Channel: Basic health centre staff in the rural districts; CHWs in the rural districts; private franchised providers in the urban areas. The distribution model used existing distributors and NGO sales staff to reduce training and operational costs.
Target population	Targeted approximately 15,000 children 6–23 months old
MNP schedule	Consume at least three sachets of MNP a week
SBCC	A social marketing strategy was developed around the 4Ps of marketing (product; place; price; and promotion) based on formative research. A communication strategy was used specifying key messages for caregivers and intermediaries in rural and urban areas, media channels, frequency of diffusion, and printed IEC tools. In the rural areas, CHWs promoted the product during home visits and community nutrition meetings. Recipe books and cooking demonstrations were also done, which helped mask the smell and taste of MNP that was problematic due to product quality issues. In the franchised clinics, doctors counselled mothers during initial and follow‐up visits. CHWs and doctors received different IEC tools including a flipchart for doctors; CHWs used a large poster with photos of foods that can be attached to the poster for foods consumed by the child during the day, to facilitate discussion on breastfeeding and IYCF. Key mass media and interprocess communication messages included reminders about long‐term benefits on repeated use as this was found lacking from CHW and doctors' counselling on repeated use. A survey was done to evaluate SBCC activities (*n* = 197 caregivers exposed to SBCC and 215 nonexposed). MNP use was higher when exposed to home visit/group talk by CHW (37% vs. 4%) and cooking demonstrations (42% vs. 13%). Culinary demonstrations also improved complementary feeding practices (59% vs. 39% for those not attending) as did receiving a home visit/group talk by a CHW (53% vs. 36%).
Training	CHWs received a 5‐day training on IYCF (refresher training for the majority), MNP, promotion and sales tips, monitoring tools, and water sanitation and hygiene. Doctors received 2.5 days of training on similar content adapted for their educational level. Doctors also received a detailed *Frequently Asked Questions and Answers* sheet.
Lessons learned	A sustainability element was built in from the start, with urban consumers paying five times the price as rural consumers. Sales revenues from the franchise providers were used to reinforce activities linked to the community‐based distribution, offsetting some of the operational costs. Providers needed regular retraining and perform best when a variety of nonmonetary incentives are offered. Due to continuous high investments in CHW training and frequent, close supervision, positive results were reported for sales and IYCF and MNP behaviours in rural areas. For urban areas, much depended on the individual provider's interest and motivation; sales and intake adherence were lower as providers had difficulty tracking caregivers to ensure adequate intake adherence.

a CHW, community health worker; IEC, information, education and communication; IPC, inter‐process communication; IYCF, infant and young child feeding; MNP, micronutrient powders; NGO, non‐governmental organization; SBCC, social behavior change communication; WASH, water sanitation and hygiene.

b Based on information from key informant 9.

#### 3.1.1. Free health sector: MNP have most commonly been distributed through the health sector free of charge as part of IYCF programmes

Under a free model, where the MNP was free to the client, MNP were usually distributed through the national health sector. The specific type of health platform has varied: Generally, MNP interventions have been incorporated into routine health facility visits (e.g., Kyrgyzstan [CDC & UNICEF, 2013] and Peru [Creed‐Kanashiro, Bartolini, Abad, & Arevalo, 2015]), a combination of health facility and/or community outreach (e.g., Cambodia [Helen Keller International, 2015; KI 5] and Nepal [Jefferds et al., 2015; KI 12; see Figure 1]), and/or use of biannual maternal and child health days (e.g., Nigeria [Korenromp et al., 2015]). Seventy‐four percent of the 59 MNP interventions reported in the most recent UNICEF NutriDash Global Report were part of the health sector's IYCF programmes (2015). IYCF programmes provide access to the appropriate target age group and mutually beneficial messaging and counselling opportunities (KIs 4, 9, and 12). Although this appears efficient, IYCF programmes have often been poorly implemented (Kim et al., 2015; UNICEF, 2014; Vanchinkhuu, Norov, & Bat, 2013), and without strengthening local implementation, adding an MNP intervention to this platform may not be effective. For example, in Kenya, weak mother‐to‐mother support groups (a platform used to promote IYCF) also reduced caregivers' participation in the MNP intervention (GAIN, n.d.; KI 10). In general, the biggest challenge using a free health sector delivery strategy raised across key informants was the uncertainty of sustained funding (Table 2).

Coverage among free health sector MNP interventions ranged from 32% to 83% (9 programmes, variable definitions) and generally was higher when distribution took place at the community level (Table 3). Nigeria, using the biannual maternal and child health days, had the lowest coverage, although the coverage for MNP was comparable to the other interventions also provided under this platform (Korenromp et al., 2015). The highest reported MNP coverage was in Cambodia and Nepal (Box 2) where MNP was integrated into IYCF programming and provided by both health facility workers and CHWs (Helen Keller International, 2015; Jefferds et al., 2015). Intake adherence ranged from 35% to 88% (5 programmes, variable definitions) across interventions delivering MNP for free through the health sector (Table 3).

#### 3.1.2. Free nonhealth sector: Well‐established nonhealth platforms have provided opportunities to reach vulnerable populations with MNP free of charge

Nonhealth sector platforms have the potential to enhance the reach of the nutrition intervention to populations beyond that of the health sector (Ruel, Alderman, & Maternal and Child Nutrition Study Group, 2013). In many cases, these programmes have extensive targeted coverage of vulnerable populations; for example, national social protection programmes in Ethiopia, Mexico, Brazil, and Ecuador reach the poorest 10% to 40% of the population (Ruel et al., 2013). Key informants thought that interventions under this platform worked best when nutrition‐specific objectives and actions were added as this facilitated the implementation of counselling necessary for successful programme outcomes (KI 8).

Evidence on delivery of MNP through nonhealth delivery is limited (Ruel et al., 2013). Examples of nonhealth sector delivery are found in social protection programmes in the Dominican Republic (World Food Program, 2015) and Mexico (Bonvecchio & PROSPERA Program, 2016; KI 11; Box 3 and Figure 1), early childhood development (ECD) programmes in Nicaragua (Lopez Boo, Palloni, & Urzua, 2014), as part of the Integrated Child Development Services Scheme in India (Fernandez‐Rao et al., 2014), and an integrated nutrition–agriculture programme in Nepal (Osei et al., 2015). Although the data are limited and definitions varied, at least some free nonhealth sector delivery interventions have achieved high coverage (ECD 75%, 1 programme; social protection 93%, 1 programme) and intake adherence (ECD 70–84%, 2 programmes; social protection 78% urban and 81% rural, 1 programme; Table 3).

#### 3.1.3. Full‐cost, subsidized, and mixed models: Increasingly fee‐based models are being considered with the intention of lowering intervention costs and building sustainability

As of 2014, 17% of the 59 reported MNP interventions in the UNICEF NutriDash Global Report were provided for a fee, ranging from subsidized to full‐cost (UNICEF, 2015). In a full‐cost MNP model, commercial entities market MNP, often as part of a group of health products, with the aim of recovering costs and/or making a profit. It has been suggested that a full‐cost model will fail to reach the poorest segment of the population and very few countries have tried this model (e.g., South Africa [KI 9]) because most contexts cannot meet the conditions required to sustain the model (Bahl et al., 2013; GAIN, The Global Business School Network, & The Tuck Global Consultancy Program, n.d.; KI 9). Subsidizing the cost of MNP has been viewed as a more viable option for partial cost recovery (Bahl et al., 2013; KI 9). In some instances, there has been agreement to reinvest profits earned from those who can pay to reach lower income groups with additional distribution/promotion activities (e.g., Madagascar [KI 9]), Vietnam (Nguyen et al., 2016; [KI 10]). Bangladesh's MIYCN Home Fortification Programme has been able to achieve ongoing sales, having sold approximately 3.9 million sachets with 1.3 million children having ever used MNP, and 48,000 children having completed 60 sachets over 6 months based on data from June 2016 (GAIN/BRAC, 2016).

There are also examples of mixed models where a free and a fee‐based product coexist, or price differentials for different segments of the populations were created, as highlighted in the Madagascar case study (Box 4 and Figure 1) (KI 9). In Nairobi County, Kenya, a mixed model was adopted part way through the programme because poor infrastructure and long travel distances hindered access to health facilities and disrupted the frequency of outreach services (GAIN, n.d.; KI 10). Adding a subsidized option to the programme design in which retail outlets offered MNP and/or MNP were sold door‐to‐door helped increase access when the free model alone was not able to achieve sufficient coverage (KI 10). Key informants raised the issue that having access to both a free and a fee‐based product under the same brand can impact consumer's perception and is a disincentive to private investors (KI 10). For example, in Lao PDR, the same product was available for free through the public health centres and social marketed for a cost via pharmacies, retail outlets, and community outreach. The field staff reported that caregivers perceived the MNP to be of low quality because it was being made available for free, and sales staff reported that retailers were reluctant to buy a product that was free in nearby locations (PSI Laos, 2015; KI 7).

Overall, although a fee‐based MNP model shows promise, it may not be affordable to the consumer in many contexts even when substantially subsidized (KI 9). The ability to maintain equitable access is especially important in the context of an MNP intervention because poorer households are generally at greater risk of anaemia (Balarajan, Ramakrishnan, Ozaltin, Shankar, & Subramanian, 2011). A few studies have examined purchasing patterns in relation to wealth using a subsidized model and have found mixed results. In Bangladesh, income was unrelated to ever having purchased MNP but higher income households had greater awareness of MNP and purchased an increased number of sachets (Rawat, Saha, Kennedy, Ruel, & Menon, 2013). In a follow‐up study, once the programme had matured, however, frequency of use was similar across “poorest” and “richest” quintile households (Angdembe, Choudhury, Haque, & Ahmed, 2015). In western Kenya, evidence showed no association between income and the number of sachets purchased, ever used, or recently used (Harris et al., 2012; Suchdev et al., 2010); several years into the intervention, poorer households were more likely to have recently consumed MNP (Suchdev et al., 2013). In Vietnam, the number of sachets purchased at one time was positively correlated to wealth and across all quintiles households were more willing to purchase a monthly package of 10 sachets, as a larger box of 60 sachets appeared as too high a one‐time cash outlay for a new product (Nguyen et al., 2016; KI 10).

No coverage data were available for the full‐cost model. There was a wide range of coverage using subsidized models (9% to 86%, 8 programmes, variable definitions, Table 3). Data from multiple years were available in western Kenya and showed high initial coverage but a steady decrease by year three of the programme (Table 3) (Suchdev et al., 2013). The definition for intake adherence was too variable to provide a range (see Table 3 for data by individual programme). Data identified for mixed models only included information on the delivery of MNP provided at one price point, so it was not possible to compare indicators for a delivery model with mixed price points (i.e., data were collected for one delivery arm but not both).

#### 3.1.4. Delivery channels: Health workers often distributed MNP irrespective of the model or platform

Distribution of MNP at health facilities followed by community distribution have been the most common delivery channels to date (UNICEF, 2015). Distribution of MNP by both health facility workers and CHWs was piloted in Cambodia and Nepal (Helen Keller International, 2015; Jefferds et al., 2015; KI 5). Both pilots used a free‐of‐charge model and an IYCF health sector platform. In the Cambodia pilot, districts could self‐select a health facility worker or CHW distribution channel (KI 5). The districts delivering MNP using a community distribution channel had higher coverage, which led some districts providing MNP at facilities to add this channel part way through the pilot (KI 5). Likewise, in the Nepal pilot, CHW distribution was found to be more successful (Jefferds et al., 2015; KI 12). Caregivers reported MNP to be more easily accessible, and this translated into a higher percentage of caregivers obtaining their first and second batch of MNP in the community compared to the health facility arm (Table 3) (Jefferds et al., 2015; KI 12). In Bangladesh, adding a CHW arm (where MNP were sold door‐to‐door) to a programme where subsidized MNP were initially provided through pharmacies resulted in an increase in MNP purchases and the ability to reach lower income households (Afsana, Haque, Sobhan, & Shahin, 2014; Rawat et al., 2013; KI 6).

Other MNP delivery channels, such as pharmacists, mobile vendors, and kiosk retailers have also been used, most commonly under a fee‐for‐product model. Although there is limited experience with these channels, in some instances, they have been found to be well accepted and feasible, such as in Niger, where community members trusted pharmacies to have nonexpired products and keep a stable and fair price (Tripp et al., 2011). However, concerns have been raised that such alternative channels may compromise the quality of counselling and follow‐up, product movement, and contribute to the difficulty of monitoring programme indicators (KI 9). To address this, in Vietnam, the chosen delivery channel was the health centre's pharmacy, where the health staff was selling the product at cost only once the mother had been consulted on basic IYCF topics and how to use the MNP (Nguyen et al., 2016). This allowed both quality counselling and financial viability of the model over time (KI 10). In Mozambique, an innovative solution was to use an electronic monitoring system of voucher redemption, where health workers at facilities distributed vouchers and the product was redeemed at a neighbouring kiosk (PSI, n.d.; KI 9).

Increasingly, health workers are used to deliver MNP irrespective of the model or platform. This is illustrated in Figure 1 showing the use of health workers in a free health platform in Nepal (KI 12), a free nonhealth sector platform in Mexico (KI 11), and a subsidized health platform in Madagascar (KI 9). In some countries, the sale of products to children through health services contravened the free health care policy. This was the case in Tanzania where all public health services to children under 5 are supposed to be free; therefore, health workers only provided caregivers information on the importance of MNP and then referred them to small stores/kiosks and pharmacies to purchase subsidized MNP (KI 3).

Despite the success of health workers (especially CHWs) as the primary MNP delivery channel, the addition of MNP to existing workloads has been considered to risk overburdening health workers (KIs 5 and 12). In Haiti and Nigeria, there was some resistance to adding MNP to the package of services offered by health workers; in the former case, the evaluators reported that this may be the result of the newness of the intervention (Loechl et al., 2009), and in the latter, the initial resistance appeared to subside after distribution activities began (Korenromp et al., 2015). Finally, an MNP pilot in Nepal found that CHWs were more likely to report needing additional support and/or disliking the workload compared to health facility workers, which led to the continuation of both health facility and community distribution postpilot (Jefferds et al., 2015).

#### 3.1.5. MNP schedules: Different MNP schedules have been efficacious, but little is known about which schedule works best across delivery channels and settings

A fixed schedule is when MNP are taken according to a prescribed frequency (e.g., daily or every other day), whereas, using a flexible schedule, caregivers are given the option of choosing their own intake frequency, as long as a fixed number of sachets are consumed within a given time period. Until recently, the WHO recommended using a fixed schedule, whereby, MNP are provided daily for a minimum of 2 months, followed by a period of 3–4 months off supplementation, so that use of the MNP is started every 6 months (WHO, 2011). However, more recently, the WHO has broadened this recommendation to a “programme target of 90 sachets/doses of MNP over a 6‐month period” to allow for the use of a fixed or flexible regimen (WHO, 2016).

A fixed scheme, derived from early MNP efficacy studies, has shown to be effective (De‐Regil et al., 2013), and more recently, the efficacy of a flexible schedule has also shown favourable results (de Pee et al., 2007; Hirve et al., 2013). A study in Bangladesh found daily versus weekly MNP reduced iron deficiency and anaemia with no difference between the study arms (Hyder, Haseen, Rahman, Tondeur, & Zlotkin, 2007), whereas in another study in Bangladesh, a flexible schedule (where the caregiver decided the frequency of providing MNP) was more efficacious than daily MNP (Ip, Hyder, Haseen, Rahman, & Zlotkin, 2009). In Lao PDR, a daily and twice weekly schedule were both efficacious although anaemic children recovered faster in the daily group (Kounnavong et al., 2011).

Two studies have found that overall intake adherence was higher among those receiving the flexible schedule in Lao PDR (Kounnavong et al., 2011) and Bangladesh (Ip et al., 2009). A follow‐up study in Bangladesh, where CHWs sold MNP, found that after shifting from an every other day to a daily schedule, mothers preferred the daily schedule (Angdembe et al., 2015). In Nepal, using a fixed daily regime with an on/off schedules may have made it difficult to re‐establish the routine of taking MNP and likely affected the programmes ability to sustain high intake adherence (Mirkovic, Perrine, Subedi, Mebrahtu, Dahal, & Jefferds, 2015a).

### 3.2 SBCC

Developing the SBCC component of an MNP intervention, as with all interventions that require behaviour change, usually includes conducting formative research (as discussed in the planning paper of this series [Schauer et al., 2017]), identifying barriers and motivators to behaviour change, and designing and implementing actions to support and encourage positive behaviours (The Manoff Group, 2012). Practice‐based experiences indicate that SBCC is essential for MNP interventions and although it is often considered at the conceptualization stage maintaining SBCC during delivery is often poorly implemented (KIs 4 and 12). The SBCC component of an MNP intervention is often delivered as a part of broader IYCF SBCC efforts for children 6–23 months of age just as the MNP itself is often delivered as part of a IYCF program through the health platform (UNICEF, 2015). However, key informants emphasized that SBCC must support MNP and IYCF practices regardless of model, platform, or channel used (KIs 4, 9, and 12).

#### 3.2.1 An SBCC strategy provides a framework to support meeting MNP behaviour objectives

An SBCC strategy provides a framework to support caregivers to meet MNP behaviour objectives. On the basis of the literature and key informants, we have identified these behaviours as obtaining MNP, sustaining appropriate use and high intake adherence, and reinforcing or even boosting other IYCF‐related behaviours (Afsana et al., 2014; de Pee et al., 2007; Suchdev et al., 2010; KIs 4, 9, and 12). We found that the need for advocacy, social mobilization, or behaviour‐focused communications is most effective when focusing on where motivation is required to lower or remove barriers or resistance to appropriate MNP use and intake adherence. Although the main focus of the strategy is on factors influencing the critical behaviours of caregivers and their influencers, the broader social and political context also needs to be considered (KI 4). For instance, in Kenya, changes in policies whereby MNP procurement shifted from central to county level caused a disruption in MNP procurement. Focused advocacy for the timely procurement of MNP was required (KI 10).

Partnerships with social marketing companies or professional communication agencies can facilitate creative and consumer‐centred communications and may help overcome costs (e.g., Bangladesh [KI 6], China [Sun et al., 2011], Madagascar [KI 9], and Vietnam [Nguyen et al., 2016; KI 10]). For instance, in China, a soybean‐based MNP—*Ying Yang Bao*—was developed in a public–private partnership (Sun et al., 2011); the government implemented the SBCC whereas a private company undertook the marketing activities through retail outlets.

Country examples of SBCC strategies and lessons learned from the literature are summarized in Table 4. In these country examples, evaluations of the SBCC strategies generally indicated high awareness and knowledge of appropriate MNP use, but data were limited. Key informants highlighted the importance of evaluating the SBCC strategy regularly and adapting it in accordance with monitoring data (KIs 4 and 12).

**Table 4:** Country examples of social behavioural change communication for micronutrient powder interventions^a^

Country	Strategy formation and activities	Evaluation of strategy	Lessons learned
Cambodia (Helen Keller International, 2015)	• Formative research done; strategy included broader IYCF messages. • Interpersonal communication by CHWs • Cooking demonstrations, TV, radio. • Materials: counselling cards, leaflets, posters.	• Baseline (2012) and endline survey (2014) (*n* = 800, *n* = 793). • Household heard about MNP: 10% to 85%. • Handwashing with soap before feeding child: 39% to 60%, no change in semisolid food or ≥4 food groups. • Caregivers reporting advised on MNP frequency (30% to 66%); correctly adding to food (20% to 54%); using 1 sachet per child per day (50% to 60%).	• Better strategies to communicate appropriate MNP use and other IYCF behaviours needed (e.g., families still mixed MNP into family's food, feeding guidelines at different ages is complex). • Explaining side effects during counselling critical. • Assessing CHWs knowledge and motivations can improve programme.
China^b^ (Sun et al., 2011)	• Formative research done; strategy included broader IYCF messages. • Interpersonal communication by doctors. • TV, newspaper. • Materials: handbooks, booklets, brochures.	• Baseline (2008) and endline survey (2010) (*n* = 250, *n* = 267). • Knew about MNP: 60%. • Appropriately mix MNP: 38%. • Minimal acceptable diet (42% to 74%), minimal dietary diversity (58% to 74%), consume iron‐rich foods (19% to 57%).	• MNP added to boiled water frequently likely because powdered soymilk mixed with water in China. • Important to use a combination of mass media, counselling, and private sector advertising; however, sensitivities around social marketing could be a challenge.
Kenya (Suchdev et al., 2010)	• Formative research done; strategy did not include broad IYCF messages. • Interpersonal communication by vendors • Skits/songs, demonstrations, road shows, loud speaker. • Materials: Cups, calendars, brochures, stickers, T‐shirts.	• Survey (*n* = 451), 12 months after distribution. • 70% received a calendar, 38% received free MNP at launches, and 19% received free MNP at training sessions. • 98% reported having heard about MNP, from vendors (49%), promotional launches (30%), and training sessions (27%).	• Monitoring showed the need for more training sessions and launches (especially in rural areas) and for revision of the incentive strategy (providing free MNP undercut sales). • Calendars perceived as the most valuable to caregivers and T‐shirts to vendors.
Lao PDR (PSI Laos, n.d.)	• Formative research done; strategy included broader IYCF messages. • Interpersonal communication by doctors. • Mobile video units and promotion at marketplace, radio, TV. • Materials: Brochures, stickers, t‐shirts.	• Interviews with 48 users and nonusers who had heard of MNP. • 60% banners and stickers, ~50% mobile video or promotional event, ~50% TV Ad, 31% radio, ~50% received brochure, 15% heard about MNP from peers, 10% heard about it from doctors.	• Banners/stickers especially effective in increasing awareness and TV ads in increasing trust. • Counselling by doctors increased trust although quality of counselling poor. • Marketplace promotion and mobile video units supported initiating use. • Cooking demonstrations effective to learn appropriate use while peers and packaging less so. • Reminder needed to continue buying.
Nigeria (Korenromp et al., 2015)	• Formative research done; strategy included broad IYCF messages. • Interpersonal communication by HWs and IYCF support groups. • Cooking demonstrations, dance, folksong, and dramas, road shows, rallies, town announcers. • Materials: manual, posters, pamphlets, flyers.	• Two surveys of caregivers attending distribution events and two home visit surveys (*n* = 896, *n* = 237, *n* = 129, *n* = 578). • Caregiver knew about MNP (83–98%). • Caregivers identified: foods MNP should (76–94%) and should not be mixed (70–89%), portion of food added to (88–92%), not adding to cooking/hot foods (81–92%). • MNP posters in facilities (56–81%), HW distributed materials: (44–57%), HW gave ≥1 group talk (69–73%), reminded caregivers how to use MNP (73–94%), tell when come for next batch (11–56%).	• Poor supply chain management for SBCC materials. • Need to include MNP dosage and pictorial instructions on the sachet. • Increased resources and better planning with local health staff needed to for social mobilization. • Social marketing and/or involving community platforms beyond MNCHW structures may save costs and increase community involvement.
Vietnam (Nguyen et al., 2016)	• Formative research done; strategy included broader IYCF messages. • Interpersonal communication by HW. • Materials: posters, educational fans, product displays.	• Survey (caregiver *n* = 962, health staff *n* = 120), 5 months after distribution. • Heard of MNP: 30% (72% among those who visited health centre). • HWs gave counselling (81%) and used counselling cards (73%), training manual (70%), educational fan (65%), educational poster (57%).	• HWs and the production of the product by the National Institute of Nutrition increased the trustworthiness of the product. • Formative research helped design a relevant brand and attractive packaging which allowed daily/monthly/semesterly dosage. • Continuous investment in SBCC is necessary to sustain effective coverage.

a CHW, community health worker; HW, facility health worker; IYCF, infant and young child feeding; MNCHW, maternal and child health week; MNP, micronutrient powders.

b Soybean‐based MNP.

#### 3.2.2 Facilitators and barriers to meeting MNP behaviour objectives have been identified across contexts

SBCC activities have been found to be most effective when informed locally by formative research (Fabrizio, van Liere, & Pelto, 2014; KI 4). Formative research to determine acceptability and feasibility, as well as develop MNP packaging and messaging, acceptability, and feasibility, is discussed in the planning paper of this series (Schauer et al., 2017). There are some common barriers and motivators to appropriate MNP use and intake adherence that have been found across contexts. Facilitating factors frequently reported included ease of use and perceived benefits (namely, increase in child's appetite, activity level, weight gain, and improved immunity), whereas inhibiting factors included a child refusing the food with the MNP added, perceived changes in texture/taste/smell of foods in which MNP were added, lack of availability of semisolid food, side effects (change in colour and consistency of stool; vomiting), and superstitions and disbeliefs about the value and benefits of the product (Angdembe et al., 2015; de Barros & Cardoso, 2016; de Pee et al., 2007; Jefferds et al., 2015; Kodish, Rah, Kraemer, de Pee, & Gittelsohn, 2011; Korenromp et al., 2015; Mirkovic, Perrine, Subedi, Mebrahtu, Dahal, Staatz, & Jefferds, 2015b; KIs 4, 9, and 12).

The frequency and quality of contact between the caregiver and the MNP channel distributor, whether it be CHWs in Bangladesh or facility health workers in Peru, has been positively associated with supporting facilitating factors and overcoming barriers (Afsana et al., 2014; Angdembe et al., 2015; Bonvecchio et al., 2007; Creed‐Kanashiro et al., 2015; Rawat et al., 2013). For instance, in India, counselling on side effects was particularly important for intake adherence (Avula, Frongillo, Arabi, Sharma, & Schultink, 2011). In Nepal, supporting mothers to establish a routine for the child's MNP intake included receiving a reminder card, and provision of this card resulted in increased intake adherence (Mirkovic et al., 2015b).

#### 3.2.3 Supporting MNP behaviours has not been found to negatively impact broader IYCF objectives

Initial concerns that promoting MNP‐specific behaviours may detract from other important IYCF behaviours have largely not been demonstrated (WHO, 2011; KI 12). Some pilots have even found improvements in the implementation of IYCF activities and broader IYCF behaviour objectives when MNP were integrated into IYCF programmes. In Vietnam, the number of IYCF counselling topics increased when the counsellors were also giving information about MNP (Nguyen et al., 2016). In Nepal, MNP distribution was associated with increased minimum dietary diversity, minimum acceptable diet, minimum meal frequency, and continued breastfeeding at 2 years of age (Mirkovic et al., 2015b). Similarly, in Madagascar, caregivers reported a positive impact on food diversification practices (PSI Research Division, 2014), and in China, meeting a minimal acceptable diet and the prevalence of consuming iron‐rich food were significantly greater by follow‐up compared to the baseline survey following introduction of soybean‐based MNP (Sun et al., 2011). Nevertheless, in Cambodia, some caretakers thought that MNP could replace quality complementary foods, highlighting the importance of framing MNP behaviours within other IYCF related behaviours (Helen Keller International, 2015). Studies have not been designed to differentiate whether adding MNP as part of an IYCF programme motivates caregivers to improve IYCF behaviours or if the generally positive impact seen is the result of strengthening IYCF programmes.

### 3.3 Training

#### 3.3.1 Continuous training is an essential component of MNP distribution and communication

Training of front‐line workers to ensure MNP distributors are providing clear and consistent messages appears critical to supporting MNP behaviours but documented lessons learned around MNP‐specific training are almost non‐existent. Key informants mentioned content for training could be standardized across settings and then later adapted to local context and the type of delivery channel (see below; KI 10). Most importantly, key informants felt that MNP training should generally be an add‐on to ongoing training on IYCF for programmes focused on children 6 to 23 months when MNP activities are framed within IYCF programming (KIs 4 and 6). In Bolivia, where MNP replaced a national iron syrup supplementation programme, inadequate initial training of health staff on providing counselling and follow‐up support to caregivers negatively impacted the intervention's acceptance (Schauer, Harding, et al., in press). In Vietnam (Nguyen et al., 2016) and Cambodia (Helen Keller International, 2015), MNP training was integrated into existing IYCF and water, sanitation and hygiene activities from the beginning; use of IEC materials and cooking demonstrations during these training sessions were effective in transferring knowledge on MNP and these broader topics (KIs 5 and 10).

Experiences in Kyrgyzstan (Lundeen, Imanalieva, Mamyrbaeva, & Timmer, 2013; KIs 1 and 9) and Madagascar (Gittelsohn & Cristello, 2014) emphasize that training for MNP interventions should include skills to clearly communicate the benefits and possible side effects of MNP, as well as alerting distributors to possible product quality issues. In Kenya, a process evaluation of an MNP intervention found that inadequate training of CHWs and programme staff on the health benefits of MNP led to decreasing intake adherence over time (Kodish et al., 2011). In India, training on diarrhoea incidence was included to help mothers understand hygiene versus MNP‐related side effects (Avula et al., 2011). Some felt that training should find a balance between informing but not overburdening the health staff (KI 9).

Programme implementers have learned that training is not a one‐time or start‐up event, especially in areas of high staff turnover (KIs 2, 5, 8, 9, 10, and 11). Ongoing training has allowed for the exchange of information and problem solving. In the Madagascar pilot, follow‐up meetings with CHWs 6–8 weeks after training provided an opportunity to share and incorporate operational issues seen in the communities (KI 9). Refresher trainings are reportedly held monthly in Bangladesh (KI 6) and were held every 3 months in Mongolia (World Vision Mongolia, 2005) when each programme was being implemented at scale.

Key informants reported that training can be costly and time‐consuming. Adding on to existing training programmes was considered a successful strategy in Madagascar (KI 9) and the Dominican Republic (World Food Program, 2015). In Bangladesh, refresher trainings were used as opportunities to resupply health workers with MNP, both to incentivize the training and to save costs. As programmes scale up and budgets are limited, concerns about maintaining the quality of training has been noted (KI 9).

#### 3.3.2 Training may need to vary based on the delivery channel

Training has needed to be tailored to the type of staff distributing MNP, which can vary greatly. For instance, Mexico's social protection programme required continual training of the roughly 75,000 health professionals and 37,000 community workers distributing MNP (Bonvecchio & PROSPERA Program, 2016; KI 11). Training content needed to be adapted multiple times to reflect the diversity of the population served (rural, urban, and indigenous) as well as the diversity of the cadres for training with their relative capacities and education levels (medical doctors, nurses, health promoters, and communicators), see Box 3 for more details (Bonvecchio & PROSPERA Program, 2016; KI 11). Although not all programmes can invest in such extensive training models, there are costs savings in online training systems, where technology allows and the number of trainees is very large.

Training of private sector staff, such as pharmacists and retailers, has been complicated in some areas as they are largely not considered accountable to a programme and are typically less motivated to provide quality counselling (KI 9). Likewise, specific training has been needed for health staff when a fee‐for‐product model is used. Training on sales and promotion techniques was reported to be necessary to enable health workers to be more successful in selling product in Madagascar, despite the built in financial incentive to sell (KI 9). Similarly, health workers needed training to make a sales‐pitch, convince a potential interested consumer in making the first “trial” purchase, and obtain loyal regular users. Such training has included interpersonal communication skills and tailoring messages on the basis of the transtheoretical model of change (Prochaska et al., 1994; KI 9).

## 4 DISCUSSION

Evidence is still needed on the best ways to deliver MNP to young children, including how to increase demand and support appropriate use and intake adherence to achieve nutritional impact in real‐world settings. In this paper, we documented experiences in MNP delivery, SBCC, and training from diverse contexts in low‐ and middle‐income countries. This paper is based on the MNP implementation literature and experts' experiences and learning. The review is not exhaustive, as our data sources were limited to published or grey literature or our ability to identify and interview someone familiar with an intervention. In addition, the information from key informants should be treated as expert opinion. As such, we cannot make broad scale inferences and it should be recognized that other stakeholders involved in the implementation of MNP might have differing experiences or viewpoints and these experiences may not be applicable across all contexts. Experiences from national or large programmes running at scale were sought, but information was mainly found for pilots, and even in these instances, documentation of programme experiences was often not comprehensive. Thus, our results should be interpreted with this caveat and highlights the need for better documentation of programme experiences during pilots and continued learning during scale‐up.

MNP interventions have primarily used a free‐of‐charge model with distribution through the health sector platform as part of IYCF. Although integration of MNP into IYCF or other health platforms can facilitate continuity and sustainability, competing priorities for countries with high disease burden or limited capacity and resources may result in weak implementation if the underlying platform is already weak. A free model using a nonhealth sector platform has shown great promise in providing MNP to large segments of the vulnerable population, most notably in Latin America through social protection programmes. We found only a few examples within the agriculture sector and early child development. The sustainability of delivery strategies where a free model has been used has been an issue, with the majority of countries using this model still reliant on external funding. This—coupled with the realization that MNP can be promoted and sold for a fee when there is strong SBCC to accompany them—has led to an interest in adopting a fee‐for‐product model using subsidy schemes.

Under a subsidized model, programmes have operated under the premise that there is the potential to extend coverage to the less vulnerable whereas simultaneously improving overall market equity and efficiency although the results on the extent of this are not clear (Afsana et al., 2014; Siekmann, Timmer, & Irizarry, 2013; Suchdev et al., 2012). We found very few examples of a full‐cost recovery MNP model potentially because of the high start‐up costs and the need for a strong and well‐developed market to sustain it, making subsidized models a more viable option in most contexts. Some successes—but mostly challenges—have been achieved using a subsidized model where fee‐based MNP coexisted with free distribution or price differentials were created for different populations. The coverage of a subsidized model has generally been lower than a free model even though markets have the potential to expand reach; continuing to learn from the private sector and apply social marketing techniques may improve programme outcomes, but this has yet to be seen. Further research is needed to examine how pricing affects purchase of MNP over the long‐term by income group, especially in association with increase in awareness and availability of the MNP.

We identified diverse experiences with MNP interventions, with some countries having multiple experiences. There was no one‐size‐fits‐all strategy even within a single country context. Delineating delivery strategies by model, platform, and channel, we found that counter to the traditional perspective of a divide between free and fee‐for‐product models, health sector and nonhealth sector platform, and types of delivery channels, there were examples of using a range of combinations. The delivery strategy definitions presented here could be adopted more widely to better reflect the emerging combinations of MNP delivery strategies.

A single delivery model, platform, or channel may not be sufficient due to variations in infrastructure, resources, distances, health burden, or other contextual factors. Community‐based distribution, especially as part of other IYCF services, whether MNP are free or subsidized, may be the most accessible for families and provide an opportunity to provide counselling on related IYCF behaviours. There have been some instances of health workers reporting an increased burden so using multiple distribution channels and access points may at least partially address this concern (Jefferds et al., 2015).

It was difficult to compare and contrast programme success due to differences in reporting indicators and variations in context‐specific factors. In this paper, we defined success as >70% for coverage and intake adherence. We found that programmes were not able to reach this threshold. This is not surprising given the coverage of other IYCF practices (e.g., breastfeeding, minimal meal frequency, and dietary diversity) among children 6 to 23 months have been found to be similarly low (UNICEF, 2015). There is not yet an agreed‐upon threshold for what constitutes programme success. Ideally, thresholds should be based on the levels that are sufficient to reach nutritional impact but current data on impact of MNP is mainly from randomized control trials and not from large scale programmes. A better understanding of what constitutes a minimal dose and over what time period is also needed globally.

Although the MNP delivery strategy plays a large role in the level of achievable coverage, the SBCC component of a programme focuses on behaviour objectives required to support appropriate use, coverage, and intake adherence; thus, the entire programme should be designed with this in mind. As is the case for other behavioural change interventions that require the regular consumption of a product for a long time, an inability to maintain programmatic success is common (Allen, 2002). Therefore, where SBCC has not been continuous and/or modified throughout the programme life cycle, MNP coverage has faltered (Suchdev et al., 2013). Investments in formative research, plus investments in the development of the strategy and its implementation and evaluation, are often forgotten or are not itemized leaving programmes without sufficient support once the MNP is available for distribution. Understanding and providing for these activities are as important for the full‐cost model where marketing and promotion efforts are required—upfront and continuously thereafter—as it is for free distribution.

MNP training is critical to facilitate desired MNP behaviours. Training on MNP specific elements has been designed and adopted by most programmes, with detailed content and duration varying across settings. Training has usually been added to ongoing training on IYCF for front‐line workers given the importance of framing MNP within broader IYCF messaging. Regular refresher trainings can help resolve problems encountered and are particularly important to address concerns and ideas that may be limiting use, product quality issues, dropout, and misuse. Training and supervision of nonhealth staff, such as pharmacists and retailers, can be challenging as they are not part of a routine health system and have less vested interest to perform according to expected standards. Programmatically, there is not much experience documented on how best to overcome this challenge.

Overall, we found some common lessons and key elements that are likely to be applicable across settings. An MNP intervention requires a motivated health (or other) sector, careful operational design building on formative research, coordinated SBCC across actors, an understanding of contextual and cultural factors, and the provision of sufficient, adequate, and timely information to both caregivers and intermediaries (Kodish et al., 2011). There was limited documentation of lessons learned on MNP at scale, but a few key lessons were drawn from the available literature. Using a well‐established delivery platform targeting the most vulnerable populations (e.g., Mexico) and taking advantage of a myriad of delivery strategies and inclusion of multiple partners (e.g., Bangladesh) facilitated delivery of MNP at scale. Key constraints to scaling up across settings were a lack of reliable funding, difficulty in maintaining high quality IYCF behavioural change activities, and the resource intensity required to conduct refresher training.

Implementation research on MNP interventions at scale and greater efforts to document and share research results and programme experiences is needed to further inform delivery, effective SBCC, and quality training. Priority research areas or tools/resources identified during the consultative process included the following:

- Document factors affecting the sustainability of different types of delivery strategies over a prolonged duration;
- Establish cost‐effectiveness of different delivery models, platform, and channels, where such data can be relatively easily collected and analysed;
- Assess sustained willingness to pay for MNP to determine feasibility of mixed or subsidized models, using appropriate methods (e.g., market based analyses);
- Determine if a mixed or subsidized model can maintain equitable accessibility to those at risk of iron deficiency or anaemia while remaining viable and sustainable (i.e., with acceptable profit margins to keep the private sector engaged);
- Further document experiences using a fixed or flexible MNP schedule to determine if preferences are consistent or context specific;
- Define common indicators and metrics for programme performance that are linked to nutritional impact;
- Identify ways to translate formative research into an effective scalable behaviour change strategy that can be accomplished with low burden and high quality by distributors;
- Document solutions to ameliorate common barriers (e.g., side effects) to meeting MNP behaviour objectives;
- Understand if and how adding MNP as part of IYCF programmes negatively or positively effects IYCF behaviours, such as the consistency of food offered to young children;
- Document the minimum skill set required to deliver MNP and how operations can be modified to ease time and resource constraints, including streamlining training;
- Assess the level of effort required by delivery channel providers to deliver effective IYCF behavioural change activities that include and do not include MNP as part of the IYCF package and compare cost‐effectiveness; and
- Determine how to manage the burden of adding MNP delivery to frontline staff workload, as well as testing different types of incentives to retain and motivate delivery channel distributors.

## CONFLICT OF INTEREST

The authors declare no conflicts of interest.

## CONTRIBUTIONS

IR and SMLN did the primary drafting of the paper with substantial contributions from AB, AP, CND, MG, MEJ, and RR. DC, HK, MEJ, MRH, and AB drafted and/or provided information on country experiences and case studies. RR chaired the WG, and SMLN served as the secretariat. All authors contributed to the conception and design of the paper and reviewed and approved the submitted manuscript.

## Supporting information

Data S1 Working Group 2 Questionnaire on delivery, social behavioural change communication, and training (as provided to key informants and/or used as interview guides)
