# Supplementary material for: Experiences and lessons learned for delivery of micronutrient powders interventions
Source: Matern Child Nutr. 2017 Sep 29;13(Suppl 1):e12495. doi: 10.1111/mcn.12495 (PMC5656897; doi:10.1111/mcn.12495)
Supplement: Supplementary file 1 — Data S1 Working Group 2 Questionnaire on delivery, social behavioural change communication, and training (as provided to key informants and/or used as interview guides) [file MCN-13-e12495-s001.doc]

**Supplementary Material 1**

**Working Group 2 Questionnaire on delivery, social behavioral change communication, and training (as provided to key informants and/or used as interview guides)**

| **Questionnaire: Delivery, Social Behavioral Change Communication, and Training Working Group** |
| --- |
| - *Your responses to this questionnaire will be used to share your experiences and insights on micronutrient powder programming across the three working groups for the upcoming MNP Consultation. Please fill out the form by October 12.* - *Do not feel required to answer every question, but please do at least skim the entire questionnaire and focus on "specific identifiable lessons learned" -- positive or negative -- that you would like to share. If you have already been interviewed by a group, you do not need to respond to that group's questionnaire.* - *Before completing this questionnaire, feel free to solicit input from colleagues past and present and others in your country or office who you think would have useful responses. Multiple perspectives from the country or office can be rolled into one response form.*   *Name: _________________________*   - *This will only be used to ensure that we can follow up with you about any questions. We will not use your name to attribute any answers without first receiving your permission*   *In what country(ies) do you have experience working with MNP programs?*  *Please note if you have any requests for anonymity of a particular country experience.* |
| **Delivery** |
| 1. In your country context, can different delivery platforms be envisioned providing the same product (but perhaps with a different pricing structure) to different groups (targeting public sector users versus private sector users)? Is this already the case, and if so, how is this working out? |
| 1. What enabling factors were most crucial to establish an optimal delivery channel? |
| 1. What do you see as the challenges and benefits to private sector delivery, public sector delivery and a hybrid or mixed model? |
| 1. What is the best performing platform (with the highest effective coverage and adherence) for health/nutrition programs in your country for your target group (young children)? Did you consider using this platform for MNPs – why or why not? |
| 1. Is there a program platform on which MNPs are delivered in your country (IYCF, ag-nutrition, ECD, social protection)- and what are the pros and cons to delivery MNPs through this platform? |
| 1. How did you address any supply chain issues, either with private sector or public sector? What kind of problems did you encounter (specifically around distribution, promotion, forecasting etc) and how were they resolved? |
| 1. Delivery and Front line workers: Do you have an incentive system in place for intermediaries of the delivery (health workers, supply points, community volunteers)? What's been the experience with this to date? |
| 1. Training Materials: Describe the process of how the training materials were developed or adapted for the country context? How much time and depth of information on MNP was needed to adequately train the various cadres at different levels? |
| 1. Training Approach: What worked well and what did not work well in the model of training that was used? Was there follow-up of trainees such as through mentoring, refresher trainings and/or supervision? |
| 1. Training and Scale up (if applicable): Describe how training during the scale-up of the program happen? Was it a cascade training or another model? Was the training integrated with other topics? If so, please describe. |
| 1. Other: Do you have any other challenges, successes and lessons learned around delivery channels and platforms you’d like to share? |
| **Social & Behavior Change Communication** |
| 1. Formative research for SBCC: Briefly describe what, if any, formative research was done and what was done with the information? (e.g., did you just develop a report or did you also get everything else needed to develop the BC program package (training materials, SB strategy, etc).  What would you do differently next time in terms of formative research?  What was the most useful information from the formative research? |
| 1. If you have an official SBCC strategy, is it integrated into the IYCF approach or separate? What are the reasons behind as well as the pros and cons of your approach? |
| 1. BC Strategy: What were the strategies used for BC? Did your BC strategy involve interpersonal communication and if so, how to you manage and for how long did you maintain that give the intensity required? |
| 1. SBCC duration: For how long was your behavior change/promotion work planned? Where did your BC strategy start its focus on and how did the BC change over time/phases (if at all).  Do you have ‘maintenance’ messaging for longer term programs and what does it look like in terms of frequency and content. How has your messaging changed over time? |
| 1. SBCC and scale up: Were you able to maintain BC that was carried out in pilot with fidelity and quality at a larger scale? If so how did you revise the BC for larger scale (what fell out of the approach or did you have enough funds to do the same things at a larger scale)?  Any other lessons learned in moving your BC campaign and strategy to a larger scale? |
| 1. SBCC monitoring data: Did you collect any data (qualitative, quantitative, monitoring, cross sectional surveys etc) to show if the changes they expected in the participants happened? (e.g., changes in knowledge, skills, motivations, practices).  How are you tracking (if that's possible/happening) user experiences with the product? Did you collected information from positive or negative deviants (e.g., high users vs low users/quitters/non-starters)? |
| 1. SBCC lessons learned: Do you have any additional information on challenges you overcame in relation to BC work and how it was accomplished? |
